# Humanized Care from the Perception of Oncology Patients from Southern Chile

**DOI:** 10.17533/udea.iee.v39n2e04

**Published:** 2021-06-15

**Authors:** Tannia Navarrete-Correa, Flery Fonseca-Salamanca, R. Mauricio Barría

**Affiliations:** 1 Nurse, Master’s. E-mail: tannia.navarrete@uach.cl tannia.navarrete@uach.cl; 2 Medical Technologist, PhD. E-mail: flery.fonseca@ufrontera.cl flery.fonseca@ufrontera.cl; 3 Nurse, PhD. Director, Institute of Nursing. E-mail: rbarria@uach.cl. Corresponding author. rbarria@uach.cl; 4 Institute of Nursing, Faculty of Medicine, Universidad Austral de Chile. Valdivia, Chile. Universidad Austral de Chile Universidad Austral de Chile Valdivia Chile; 5 Faculty of Medicine, Universidad de la Frontera. Temuco, Chile. Universidad de La Frontera Universidad de la Frontera Temuco Chile

**Keywords:** humanization of assistance, oncology nursing, nurse-patient relations, health education., humanización de la atención, enfermería oncológica, relaciones enfermero-paciente, educación en salud, humanização da assistência, enfermagem oncológica, relações enfermeiro-paciente, educação em saúde

## Abstract

**Objective.:**

This work sought to determine the perception of behaviors of humanized nursing care and its relation with sociodemographic and clinical variables in patients hospitalized in a Hemato-Oncology Department.

**Methods.:**

Analytic cross-sectional study conducted with 51 patients hospitalized in the Hemato-Oncology Unit at Hospital Base Valdivia, Chile. A survey containing sociodemographic and clinical information was applied together with the questionnaire on *Perception of Behaviors of Humanized Nursing Care 3* ^*rd*^ *version” -PBHNC 3v* (32 items distributed in the categories: Qualities of nursing work, Openness to nurse-patient communication, and Willingness to care).

**Results.:**

Of the participants, 51% were women, with mean age of 46.5±16.6 years; 54.9% were diagnosed with Lymphoma and 78.4% were in the treatment induction stage. In 30 of the 32 items of the instrument, > 90% of the participants evaluated compliance with the behavior of caring at level of “always”. By categories, it was observed that for “Willingness to care” there was significantly lower score among patients from 18 to 49 years of age (*p*=0.0455). For the category “Openness to nurse-patient communication” lower median score existed in patients with Myeloma (*p*=0.0043) and in patients in the Remission-Consolidation stage (*p*=0.0084). Days of hospitalization were associated significantly with the category “Willingness to care”, being lower with 16 days and more (*p*=0.0242).

**Conclusion.:**

High frequency was observed of humanized-care behaviors and small differences in their assessment that were associated with demographic factors like age, and clinical factors, like diagnosis, treatment stage, and days of hospitalization.

## Resumen

**Objetivo.:**

Determinar la percepción de comportamientos de cuidados humanizados de enfermería y su relación con variables sociodemográficas y clínicas en pacientes hospitalizados en una Unidad de Hemato-Oncología.

**Métodos.:**

Estudio transversal analítico realizado con la participación de 51 pacientes hospitalizados en la Unidad de Hemato-Oncología del Hospital Base Valdivia, Chile. Se aplicó un formulario que contenía información sociodemográfica, clínica y el instrumento *Percepción de Comportamientos de Cuidados Humanizados de Enfermería 3ª versión” -PCHE 3v* (32 ítems distribuidos en las categorías: Cualidades del hacer de enfermería, Apertura a la comunicación enfermera(o)-paciente y Disposición para la atención).

**Resultados.:**

De los participantes, 51% fueron mujeres, con una edad media de 46.5±16.6 años. Un 54.9% presentó diagnóstico de Linfoma y 78.4% estaba en etapa de Inducción del tratamiento. En 30 de los 32 ítems del instrumento, sobre 90% de los participantes evaluaron el cumplimiento del comportamiento de cuidado en nivel “siempre”. Por categorías se observó que para “Disposición para la atención” hubo significativa menor puntuación entre los pacientes de 18 a 49 años (*p*=0.0455). Para la categoría “Apertura a la comunicación enfermera(o)-paciente” existió menor puntaje mediano en pacientes con Mieloma (*p*=0.0043) y en los pacientes en etapa de Remisión-Consolidación (*p*=0.0084). Los días de hospitalización se asociaron significativamente con la categoría “Disposición para la atención”, siendo menor con 16 días y más (*p*=0.0242).

**Conclusión.:**

Hubo alta frecuencia de comportamientos de cuidados humanizados y pequeñas diferencias en su valoración que se asociaron con factores demográficos como la edad y a factores clínicos como el diagnóstico, la etapa de tratamiento y los días de hospitalización.

## Resumo

**Objetivo.:**

Verificar a percepção dos comportamentos de cuidado humanizado de enfermagem e sua relação com variáveis ​​sociodemográficas e clínicas em pacientes internados em uma Unidade de Hemato-Oncologia.

**Métodos.:**

Estudo transversal analítico realizado com a participação de 51 pacientes internados na Unidade de Hemato-Oncologia da Base Hospitalar de Valdivia, Chile. Foi aplicado um formulário que continha informações sociodemográficas e clínicas e o instrumento Percepção de Comportamentos da Assistência Humanizada de Enfermagem 3ª versão -PCHE 3v (32 itens distribuídos nas categorias: Qualidades do trabalho de enfermagem, Abertura à comunicação do enfermeiro (a) - Paciente e Disponibilidade para o cuidado).

**Resultados.:**

Dos participantes, 51% eram mulheres, com média de idade de 46.5±16.6 anos. 54.9% apresentavam diagnóstico de Linfoma e 78.4% encontravam-se na fase de indução do tratamento. Em 30 dos 32 itens do instrumento, mais de 90% dos participantes avaliaram a adesão ao comportamento de cuidado no nível “sempre”. Por categorias, observou-se que para "Disponibilidade para o cuidado" houve significativamente menor pontuação entre os pacientes de 18 a 49 anos (*p*=0.0455). Na categoria "Abertura à comunicação enfermeiro-paciente" houve menor pontuação mediana nos pacientes com Mieloma (*p*=0.0043) e nos pacientes em fase de Remissão-Consolidação (*p*=0.0084). Os dias de internação estiveram significativamente associados à categoria “Disponibilidade para o atendimento”, sendo menos com 16 dias e mais (*p*=0.0242).

**Conclusão.:**

Observou-se alta frequência de comportamentos de cuidado humanizado e pequenas diferenças na avaliação que estiveram associadas a fatores demográficos como idade e a fatores clínicos como diagnóstico, fase do tratamento e dias de internação.

## Introduction

The care and wellbeing of people receiving health care seeks to be the focus of every health system. Given this, it is imperative that these be delivered by humanized systems, that is, that these involve closely the ethical commitment and caring for people. This is based on the fact that said ethical treatment, worthy and of quality, is deserved by every person and within the health environment; this setting is understood as a right, a transversal human good and a fundamental axis in society.(1) The human and therapeutic relation between patients and professionals who provide care comprises a social phenomenon that is the core of treatment success within the health-disease process,(2) and which has implications, among other aspects, on adherence to treatment, always mediated by qualities like respect, empathy, and motivation.(3,4)

Health care has become sophisticated due to progress in knowledge and technology, which - undoubtedly - has contributed to better health management, but also has generated fragmentation within care, finding patients who perceive distant care, and manifest feeling vulnerable in front of professionals with mostly technical and procedural aptitudes than with a human attitude.(5) Humanization in caring has been proposed and highlighted for some years by Jean Watson, principally because the rational dimension of nursing, that is, the affective transpersonal relationship, stands in the foreground. The patient-nurse meeting can evolve to a moment of care, depending on the conscience, authenticity, and intentionality of the professional. A relationship of transpersonal care transforms the nurse and the patient alike, or in other words, it is characterized by mutuality and reciprocity. Additionally, the nurse’s commitment seeks to help the patient to find meaning in their life and health experiences.(6)

Human care, then, includes the presence and dialogue between the care provider and the patient, and this real presence is part of the concept of environment in care. This environment provides an atmosphere that facilitates communication at individual and collective levels and is not limited to physical domain measurements.(7) Moreover, the association and humanist-scientific interaction constitute the essence of the nursing discipline, hence, it is fundamental for nursing professionals to focus on what needs to be done, from humanized care, which increases quality and user satisfaction, thus, having better resolution of their care demands, and covering integrally and globally the users’ needs.(8) The wear and vulnerability of patients with diseases of poor prognosis and with high lethality in the often limited term, make it necessary to emphasize care for these patients and their families as human beings and to efficiently and closely develop humanized care. In this sense, cancer patients are subjected to multiple factors that impact directly on their health-disease process, which condition explicit needs of humanized care that include the emotional, spiritual, social, and affective needs of a patient considered severely sick.(9)

Due to the foregoing, this work focused on delving into the experience of humanized care, so far studied mostly from the depersonalization of health professionals, or vulnerability of patients,(10) and looking for some factors that could be related with this experience. Consequently, the objective was to determine the perception of behaviors of humanized nursing care and its relation with the sociodemographic and clinical variables of patients hospitalized in a Hemato-Oncology Department of a high-complexity hospital from southern Chile, who were subjected to rigorous care at technical level, and furthermore are vulnerable patients by being closely linked to a high mortality scenario.

## Methods

Analytical cross-sectional study conducted with 51 patients from the Hemato-Oncology Department at Hospital Base Valdivia, a high-complexity establishment in the city of Valdivia, Los Ríos region, southern Chile, during the fourth trimester of 2019. This hospital constitutes a regional and supra-regional reference center for hemato-oncological disorders and radiotherapy of the country’s Extreme South Macro Zone. The study worked with target population and not with a sample, given that the statistics of hospital discharges for the fourth quarter of the previous year (2018) were reviewed, which were 52 patients. Consequently, all potential participants of the fieldwork period were invited (October to December 2019).

The inclusion criteria established were being ≥ 18 years of age, hospital stay for > 48 h, and having signed the informed consent to participate. The exclusion criteria considered any condition that implied neurological compromise or cognitive alteration, which would impede answering the questionnaire. The instruments used included a questionnaire created for this study, to obtain information from the sociodemographic and clinical setting. The study also applied the instrument Perception about Behaviors of Humanized Nursing Care 3rd version (PBHNC V3) proposed by Rivera y Triana, (11) that permits evaluating the characteristics of humanized nursing care in hospital scenarios. The adaptation, validity, and measurement of the reliability of the PBHNC v3 instrument was carried out by González in Colombia,(12) and among its psychometric tests highlights 0.98 content validity (face validity = 0.78) and internal consistency of 0.96. The instrument has 32 items designed directionally, grouped into three categories: i) Qualities of nursing work that include seven items; ii) Openness to nurse-patient communication that contains eight items; iii) Willingness to care, which encompasses the remaining 17 items. Each item is structured as a four-point Likert scale, with levels associated with the score, like: 1 never, 2 sometimes, 3 almost always, and 4 always. Thus, the instrument has a score range from 32 to 128. In Chile, the transcultural adaptation and validation of the same instrument was conducted by experts, but with a limited population (*n = 60*),(13) and, therefore, it may suffer from validity limitations. Nevertheless, this experience permitted recognizing that the questionnaire is applicable to the Chilean context. This work opted for applying the original version validated in Colombia after receiving authorization from the authors.

This study performed a descriptive statistical analysis and of association of the study variables, with calculation of mean, medians, mode and standard deviation to determine the perception of humanized care (global and by categories) provided nursing professionals to patients hospitalized in the unit described. The analysis began with an exploratory data evaluation to detect inconsistent or lost data. Thereafter, the descriptive analysis was developed from measures of central tendency and dispersion for the quantitative variables, like age, days of hospitalization, global score and score by categories, among others. Meanwhile, the qualitative, nominal and categorical variables (sex, diagnosis, treatment stage, etc.) are presented using absolute and relative frequency distribution. To describe pertinently the quantitative variables and then, determine the adequate association analysis techniques, the fit of the quantitative variables to the normal distribution was evaluated by graphic evaluation (histogram) and the Shapiro-Wilk test. Thus, means and their standard deviation (SD) are described, and medians with the interquartile range (IQR). The analysis of association compared the median scores of the global PBHNC, and scores by categories, according to categorical sociodemographic and clinical variables, using the Mann-Whitney U test or the Kruskal-Wallis test, as corresponded. A level of statistical significance (α) of 0.05 was established. All the data were registered on a chart in Microsoft Office Excel 2016 (Microsoft Corporation, Redmond, WA, USA), and then exported to the Stata v.13 statistical package (College Station, TX, StataCorp LP 2013).

In the development of the study, the ethical aspects were protected with the evaluation and approval of the protocol by the Human Research Ethics Committees from the Valdivia Health Services, 2019 (Ord. N°354), respecting the principles and norms of the bioethics of human research.

## Results

Regarding the sociodemographic profile, 51% of the group studied were women, with mean age of 46.5±16.6 years, 54.9% within the age range between 18 and 49 years; 47.1% came from the Region of Los Ríos, mostly from the urban area (72.5%). In all, 92.2% had a support network during their hospitalization period. Furthermore, with respect to the clinical profile, it was found that 54.9% were admitted due to diagnosis of Lymphoma, 78.4% was in the induction stage of their treatment, with a median of 15 days of hospitalization, and 68.6% de los patients had no previous hospitalizations (Table 1).

**Table 1:** Sociodemographic and clinical profile of the patients *(n =* 51*)*

Variable	Value
Sex; *n* (%)	
Male	25 (49)
Female	26 (51)
Age, years; Mean (SD)	46.5 (16.6)
Age range n (%)	
18-49	28 (54.9)
50 and more	23 (45.1)
Region; *n* (%)	
Los Lagos	20 (39.2)
Los Ríos	24 (47.1)
Other	7 (13.7)
Area; *n* (%)	
Urban	37 (72.5)
Rural	14 (27.5)
Has support network; *n* (%)	47 (92.2)
Diagnosis; *n* (%)	
Lymphoma	28 (54.9)
Leukemia	15 (29.4)
Myeloma	8 (15.7)
Treatment stage; *n* (%)	
Induction	40 (78.4)
Remission-Consolidation	11 (21.6)
Hospital stay, days; Median	15 (6-31)
Days of hospitalization; *n* (%)	
< 7	13 (25.5)
7-15	13 (25.5)
16-30	12 (23.5)
31 and more	13 (25.5)
Previous hospitalizations; *n* (%)	16 (31.4)

SD: standard deviation; IQR: Interquartile range.

Regarding the perception of humanized care and the assessment by patients of each category of the PBHNC v3, it was observed that the global score ranged between 94 and 128 with a median value of 128. By categories, the median values were: Qualities of nursing work 28, Openness to nurse-patient communication 32 and Willingness to care 68 (Table 2).

**Table 2:** Descriptive statistics of the PBHNC 3v scores, global and by categories in patients from the Hemato-Oncology Unit at HBV *(n =* 51*)*.

PBHNC v3	Items	Median	IQR	Minimum	Maximum
**Global score**	32	128	126-128	94	128
Categories					
Qualities of nursing work	7	28	28-28	23	28
Openness to nurse-patient communication	8	32	31-31	21	32
Willingness to care	17	68	67-68	44	68

IQR: Interquartile range.

Additionally, it is highlighted that only two items were evaluated with less than 90% of the maximum value, with these being item 12 from the category of Openness to nurse-patient communication (“They indicate their name and position before performing the procedures”), (76.5%) and item 13 from the category of Willingness to care (“They dedicate the time required for your care”), (86.3%). Besides, 18 items were evaluated entirely with the valuation of Always or Almost always (Table 3).

**Table 3:** Distribution of patients (*n = 51*) according to the PBHNC 3v evaluation.

Category/related items	Always (4)	Always (4)	Almost always (3)	Almost always (3)	Sometimes (2)	Sometimes (2)	Never (1)	Never (1)
Category/related items	*n*	*%*	*n*	*%*	*n*	*%*	*n*	*%*
Qualities of nursing work								
1. They make you feel like a person	48	94.1	3	5.9	0	0	0	0
2. You are treated kindly	51	100	0	0	0	0	0	0
6. They make you feel well cared for when they talk to you	47	92.2	4	7.8	0	0	0	0
7. They make you feel calm when they are with you	51	100	0	0	0	0	0	0
8. They generate confidence when caring for you	50	98.0	1	2	0	0	0	0
15. They explain the care using a slow tone of voice	48	94.1	3	5.9	0	0	0	0
17. They show respect for your beliefs and values	48	94.1	2	3.9	1	2	0	0
Openness to nurse-patient communication								
4. They look in your eyes when talking to you	48	94.1	3	5.9	0	0	0	0
5. They take time to clarify your concerns	46	90.2	4	7.8	1	2	0	0
9. They facilitate dialogue	46	90.2	3	5.9	2	3.9	0	0
10. They explain the procedures previously	47	92.2	2	3.9	1	2	1	2
11. They answer your questions with security and clarity	47	92.2	4	7.8	0	0	0	0
12. They state their name and position before performing the procedures	39	76.5	7	13.7	5	9.8	0	0
14. They provide indications about your care when you require it or according to your health situation	47	92.2	4	7.8	0	0	0	0
19. They provide you with sufficient and timely information so that you can make decisions about your health situation	48	94.1	3	5.9	0	0	0	0
Willingness to care								
3. They show interest in providing you with comfort during your hospitalization	50	98	1	2	0	0	0	0
13. They dedicate the time required for your care	44	86.3	6	11.7	1	2	0	0
16. They call you by your name	49	96.1	1	2	1	2	0	0
18. They care for your basic needs (hygiene, food, urinary and bowel evacuation) in a timely manner	50	98	1	2	0	0	0	0
20. They tell you that they are looking out for you	48	94.1	3	5.9	0	0	0	0
21. They allow you to express your feelings about the disease and treatment	46	90.2	3	5.9	2	3.9	0	0
22. They respond promptly to your call	47	92.2	3	5.9	1	2	0	0
23. They identify your physical, psychological, and spiritual needs	46	90.2	4	7.8	1	2	0	0
24. They listen to you attentively	47	92.2	3	5.9	1	2	0	0
25. They inquire and show concern for your mood	46	90.2	2	3.9	2	3.9	1	2
26. They provide you with warm and delicate care	48	94.1	2	3.9	1	2	0	0
27. They help you to manage physical pain	49	96.1	1	2	1	2	0	0
28. They demonstrate that they are responsible with your care	48	94.1	3	5.9	0	0	0	0
29. They respect your decisions	49	96.1	2	3.9	0	0	0	0
30. They tell you that when you need something, you can call them	51	100	0	0	0	0	0	0
31. They respect your intimacy	50	98	1	2	0	0	0	0
32. Medications prescribed by the physician are administered on time	50	98	1	2	0	0	0	0

Upon analyzing the relation of the perception of humanized care and the sociodemographic variables, it was proven that sex, area, support network, and region had no statistically significant differences regarding the PBHNC 3v global score and its categories. The age range had no statistically significant differences in the categories of Qualities of nursing work and Openness to nurse-patient communication. However, in Willingness to care significant association was established, detecting lower scores in participants between 18 and 49 years of age (Table 4).

**Table 4:** Global PBHNC 3v score and by categories (median and [interquartile range]), according to sociodemographic variables

Variables	Global PBHNC sore	Qualities of nursing work	Openness to nurse-patient communication	Willingness to care
Sex	*p=0.1210* ^*^	*p=0.9762* ^*^	*p=0.1535* ^*^	*p=0.2858* ^*^
Male (*n = 25*)	127 125-128	28 28-28	32 31-32	68 67-68
Female (*n = 26*)	128 127-128	28 28-28	32 32-32	68 68-68
Area	*p=0.4075* ^*^	*p=0.2781* ^*^	*p=0.5273* ^*^	*p=0.6003* ^*^
Urban *(n = 37)*	127 127-128	28 28-28	32 31-32	68 67-68
Rural *(n = 14)*	128 126-128	28 28-28	32 31-32	68 68-68
Support network	*p=0.2680* ^*^	*p=0.3755* ^*^	*p=0.5236* ^*^	*p=0.2118* ^*^
Yes *(n = 47)*	128 126-128	28 28-28	32 31-32	68 67-68
No *(n = 4)*	128 127.5-128	28 28-28	32 31.5-32	68 68-68
Age range	*p=0.3585* ^*^	*p=0.8693* ^*^	*p=0.9734* ^*^	*p=0.0455* ^*^
18-49 (*n = 28)*	127 126-128	28 28-28	32 31-32	68 66.5-68
50 and more (*n = 23)*	128 127-128	28 28-28	32 31-32	68 68-68
Region	*p=0.1440* ^*†*^	*p=0.0796* ^*†*^	*p=0.1927* ^*†*^	*p=0.3902* ^*†*^
Los Lagos (*n = 20)*	127.5 127-128	28 28-28	32 31-32	68 67.5-68
Los Ríos *(n = 24)*	128 127-128	28 28-28	32 31.5-32	68 67.5-68
Other *(n = 7)*	125 98-128	28 26-28	31 24-32	68 54-68

^*^ Mann-Whitney U test; ^†^Kruskal-Wallis H test

With respect to the relation with clinical variables, it was noted that the diagnosis and treatment stage had no significant differences in the categories of Qualities of nursing work and Willingness to care but did indeed in Openness to nurse-patient communication and the PBHNC global score. There was a lower score in patients with Myeloma and subjects in the Remission-Consolidation stage. Additionally, the variable of days of hospitalization was significantly associated with the category of Willingness to care, which had lower scores in those with 16 days and more. Lastly, the variable previous hospitalizations had no significant differences in relation with the global score and the categories of the PBHNC 3v instrument (Table 5).

**Table 5:** Global score and by categories of the PBHNC v3 (median and [interquartile range]) and the clinical variables

Variables	Global PBHNC score	Qualities of nursing work	Openness to nurse-patient communication	Willingness to care
Diagnosis	*p = 0.0118* ^*†*^	*p = 0.4618* ^*†*^	*p = 0.0043* ^*†*^	*p = 0.1997* ^*†*^
Lymphoma *(n = 28)*	128 127-128	28 28-28	32 32-32	68 68-68
Leukemia *(n = 15)*	128 124-128	28 28-28	32 30-32	68 67-68
Myeloma *(n = 8)*	125 111.5-127	28 27-28	30 26.5-31.5	67.5 60-68
Treatment stage	*p = 0.0144* ^*†*^	*p = 0.1579* ^*†*^	*p = 0.0084* ^*†*^	*p = 0.0707* ^*†*^
Induction *(n = 40)*	128 127-128	28 28-28	32 31-32	68 68-68
Remission-Consolidation *(n = 11)*	125 109-128	28 26-28	31 26-32	68 57-68
Days of hospitalization	*p = 0.3242* ^*^	*p = 0.0895* ^*^	*p = 0.5282* ^*^	*p = 0.0242* ^*^
≤ 15 *(n = 26)*	128 127-128	28 28-28	32 31-32	68 68-68
16 and more *(n = 25)*	128 124-128	28 28-28	32 30-32	68 66-68
Previous hospitalizations	*p = 0.9647* ^*^	*p = 0.5853* ^*^	*p = 0.7657* ^*^	*p = 0.5963* ^*^
No *(n = 35)*	128 126-128	28 28-28	32 31-32	68 67-68
Yes (*n* = 16)	127.5 126.5-128	28 28-28	32 30.5-32	68 67.5-68

^*^ Mann-Whitney U test; ^†^Kruskal-Wallis H test

## Discussion

This study permitted establishing the perception of behaviors of humanized nursing care by patients hospitalized in a Hemato-Oncology Unit, highlighting the high score assigned to said care. In addition, it has been found that some sociodemographic and clinical variables are related with the patient’s perception. In agreement with our findings, results within a similar Chilean context underscore that 86% of the patients hospitalized considered that they “always” received humanized care.(14) This same idea is held in other contexts, given that in Peru, more recently, perception was reported at excellent level in 84% of the patients from different hospitalization services (15) and in Colombia, 72% provided the same score.(16) Meanwhile, Mexico recently reported that 67% of the participants from the context of surgical hospitalization, perceived the behavior of humanized care as favorable.(17) Nevertheless, another Colombian experience reported that only a little more than half of the patients studied (54.5%) considered that they “always” perceive the humanized-care behavior.(18)

From the point of view of sociodemographic factors, our work found no association with the patients’ sex, while age was associated with the perception of humanized care in the settings included in the category of Willingness to care, where lower score was observed in individuals < 50 years of age. This coincides, in part, with that reported by Echevarría(15), who found association of the quality of humanized care with the variables of sex and age.

From the clinical point of view, days of hospitalization were among the factors associated significantly with the category of Willingness to care of the humanized-care behavior, but no relation existed with prior hospitalization. In the Colombian experience, neither days of hospitalization or the antecedent of prior hospitalization was related with the humanized-care behavior.(18) Within the same clinical context, in patients in the Remission-Consolidation stage lower frequency was evidenced in some behaviors of nursing care linked to humanized care within the setting of Openness to nurse-patient communication. Empirical observation permits supposing that this perception is given, in the first instance, by greater exposure to care and higher time of hospitalization in such a way that the care received, and the dialectical relationship established with the nursing professional is made more critical.

Whatever the scenario may be, always providing humanized care permits improving the quality of the existence of people, given that working with human sensitivity permits modifying and/or enhance positive conducts that impact upon a disease process, and confirm that the essence of nursing, that of caring, is significant and impacts upon the person when it is humanized because professionals are guided by values that will always look for the maximum wellbeing of others.

As concluded by Beltrán,(19) from a phenomenological study in cancer patients, humanized care is framed - finally - in the nurse-patient interaction, which implies demonstrating willingness and interest in the subject of care beyond the technical, considering ethical aspects and values of care and, lastly, having communications skills to establish a significant dialogic relationship with other, considering the individual characteristics and those of the environment.

Limitations and strengths. Some limitations in this work must be considered to interpret and value its findings and scope. We could recognize that, although the study recruited all the patients from a period, the non-probabilistic nature of the selection limits - in part - the work’s external validity. Moreover, we are unaware if a seasonal behavior may exist in user perception, given that the selection of the subjects occurred during a limited amount of time. Finally, the instrument applied to assess humanized care, although widely used, does not have rigorous validation in the Chilean context and although it has been already applied in our own setting, it must be recognized that the validity of the measurement could also be affected.

Notwithstanding the aforementioned, this work has strengths, like having collected the data in standard manner by the only researcher and that the analysis performed complied with the methodological rigor required giving the pertinent statistical treatment.

## Conclusion

Nursing care, aimed at providing services in humanized manner, is recognized favorably by patients. Oncology departments require suitable professionals that show skills in distinct environments of clinical performance, integrating procedural and technical aspects and those aimed at emotional support, the capacity to prioritize the care demands and the disposition to communication. This requires identify potential sociodemographic and clinical factors that could necessitate greater emphasis in the dialectical nurse-patient relationship.
